# Classification of Odontogenic Sinusitis: A Clinical‐Based Phenotyping System to Guide Multidisciplinary Surgical Management

**DOI:** 10.1002/lio2.70555

**Published:** 2026-09-28

**Authors:** Lorenzo Sabatino, Simone Di Giovanni, Michelangelo Pierri, Carmen Bigliardo, Marco Mattaroccia, Matteo Valentini, Antonio Moffa, Manuele Casale, Michele Antonio Lopez

**Affiliations:** ^1^ Department of Otolaryngology Fondazione Policlinico Universitario Campus Bio‐Medico Rome Italy; ^2^ Department of Otolaryngology, School of Medicine and Surgery Università Campus Bio‐Medico di Roma Rome Italy; ^3^ Specialized Odontostomatology Department of Head and Neck and Sensory Organs, Division of Oral Surgery and Implantology Fondazione Policlinico Universitario A. Gemelli IRCCS–Università Cattolica del Sacro Cuore Rome Italy

**Keywords:** classification system, functional endoscopic sinus surgery, maxillary sinusitis, multidisciplinary treatment, odontogenic sinusitis

## Abstract

**Background:**

Odontogenic sinusitis (ODS) is a condition that requires a multidisciplinary approach for successful treatment. Effective communication among the healthcare providers involved reduces the risk of errors and treatment failure. This study proposes a new ODS classification system to facilitate the communication of pivotal clinical information.

**Materials and Methods:**

In this observational study the authors considered 124 consecutive patients with ODS treated in the Campus Bio‐Medico of Rome Foundation Hospital. The main elements influencing the treatment of this condition resulted as: timing, etiology, presence of foreign body, maxillary obstruction of the ostiomeatal complex, and presence of oroantral fistula (OAF); these elements were organized in the acronym TEBOF. Each patient received a TEBOF classification. The “T” was categorized in “T_c_” for patients diagnosed with chronic sinusitis, “T_a_” for of acute sinusitis, and “T_0_” for patients with maxillary sinus foreign body without sinusitis. The “E” was differentiated in “E_c_” when the condition was a side effect of a primary dental pathology, “E_p_” when the initial cause was not a dental implant, and “E_a_” for when the initial cause was having a dental implant placed. “B” was reported as “B_1_” when there was a maxillary sinus foreign body and “B_0_” when there was not. “O” was reported as “O_1_” when the ostiomeatal complex resulted closed and “O_0_” when it was clear. “F” was used as “F_1_” when there was a communication, and “F_0_” when there was not. A preliminary assessment was performed considering the change in the classification in the groups of patients that underwent different therapeutic approaches. This classification provides a concise and easy‐to‐understand combination facilitating communication between the various healthcare providers who are essential for the appropriate treatment of this condition.

**Results:**

Among the 124 patients included (64 women, 51.6%; mean age 57.2 ± 13 years), chronic sinusitis (T_c_) was the most frequent timing category (96 patients, 77.4%), while classical dental pathology represented the predominant etiology (E_c_, 88 patients, 70.9%). Foreign body was detected in 39 patients (31.5%), ostiomeatal complex obstruction in 80 patients (64.5%), and oroantral fistula in 52 patients (41.9%). Combined surgical treatment (ESS + oral surgery) was the most frequently adopted approach (70 patients, 57%), followed by dental treatment alone (34 patients, 27%) and ESS alone (20 patients, 16%). The overall success rate was 97%, only four patients required second surgical procedure.

**Conclusions:**

The TEBOF classification provides a concise, reproducible, and clinically intuitive tool for the characterization of odontogenic sinusitis. By integrating timing, etiology, and key anatomical features into a compact alphanumeric code, it facilitates multidisciplinary communication between otolaryngologists and dental providers, and may guide the selection of the most appropriate surgical strategy for each patient.

**Level of Evidence:**

4.

## 1 Introduction

Odontogenic sinusitis (ODS) is an infectious condition of the paranasal sinuses resulting from dental pathology or treatment.

A dental infection or iatrogenic injuries (e.g., dental implants, oroantral communication following dental extractions and sinus floor elevation, foreign bodies) can violate the Schneiderian membrane, carrying infection and triggering a mucosal inflammatory response [1, 2, 3].

The diagnosis is often challenging due to diversified clinical presentation [4]. One of the challenges with diagnosing ODS is that patients often require evaluations by both otolaryngologists and dental providers. When otolaryngologists are first consulted, the clinical presentations can mimic nonodontogenic rhinosinusitis, and odontogenic sources may not be investigated.

According to consensus statement on diagnosis of ODS, in presence of these elements is necessary a multidisciplinary evaluation with otolaryngologist and dental provider [5]. The otolaryngologist should confirm sinusitis with nasal endoscopy by identifying unilateral purulence, oedema, or polyps in the middle meatus, or maxillary sinus. The dental provider confirms odontogenic sources of sinusitis evaluating the presence of necrotic o partially necrotic pulp or failing root canal therapy.

The multidisciplinary approach ensures a correct diagnosis and consequently an appropriate therapy. Otolaryngologists and dental providers should check for dental discomfort, oroantral fistula (OAF), pathologies, or previous treatments as well as sinusitis symptoms and dental pain. In cases with ODS otolaryngologists should typically confirm the sinusitis, and dental specialists should typically confirm the odontogenic pathology [6].

The otolaryngologist, dental provider, and patient should collaborate on management decisions and talk about the advantages and disadvantages of endoscopic sinus surgery (ESS) and dental care. When both dental procedures and ESS are carried out for ODS brought on by endodontic disease, periodontitis, permanent OAF, and dental implants, the majority of case series have reported high success rates of 90%–100% [2]. Oral antibiotics alone has been shown a low efficacy [7]. Patient with strong sinonasal symptoms and few dental complaints may benefit from primary ESS to relieve symptoms rapidly, followed by eventual any necessary dental care [8]. It is advisable to prioritize the district causing the least tolerable symptoms for the patient. ESS is often required following primary dental care either to relieve sinonasal symptoms or to prevent risk orbital or cerebral consequences of ODS [9], but also hybrid approaches such as the osteoplastic surgical approach (OPSA) to the maxillary sinus can be effective [10].

The heterogeneity of causes, clinical presentation, and treatment requires a different clinical strategy than “classic” rhinosinusitis. The literature on ODS is varied, this is due both to its clinical heterogeneity and the involvement of different professional figures [5]. The creation of uniform classification standards is necessary given the lack of defined guidelines.

To the best of our knowledge, no existing classification system simultaneously integrates timing, etiology and phenotypical characteristics, such as ostiomeatal complex status, foreign body, and oro‐antral fistula into a single, communicative code suitable for standardized multidisciplinary application.

The aim of this study is therefore to propose and validate a new classification system, named “TEBOF,” designed to provide a concise and clinically significant framework for the multidisciplinary management of odontogenic sinusitis.

## 2 Materials and Methods

A retrospective study was performed considering 124 patients visited and operated in the Department of Otolaryngology, Fondazione Policlinico Universitario Campus Bio‐Medico (Rome, Italy) from December 2020 to December 2023 and that have consented to the use of the clinical data reported in this article by signing a specific consent form.

A 3‐year retrospective analysis was conducted, considering clinical results, demographic variables (age and sex), disease etiology and progression, imaging, and surgical and medicinal interventions.

Clinical, demographic, and radiological data were retrospectively retrieved from medical records. Each patient's clinical profile was subsequently processed and assigned a TEBOF classification by the study team. A minority of patients presented without overt sinonasal symptoms but were found to harbor a foreign body within the maxillary sinus on CT imaging. Although this constitutes an atypical clinical scenario that does not strictly satisfy the diagnostic criteria for odontogenic sinusitis, these cases were systematically recorded in our institutional database within the context of odontogenic sinusitis management and were therefore included as a distinct subclass (*T*_0_) of the TEBOF classification, given their well‐documented potential to evolve into clinically overt disease.

### 2.1 Inclusion Criteria

1. Diagnosis of odontogenic sinusitis established through multidisciplinary evaluation by both an otolaryngologist and a dental provider, in accordance with the consensus diagnostic criteria proposed by Craig et al., including nasal endoscopy identifying unilateral purulence, edema, or inflammatory changes in the middle meatus or maxillary sinus, and dental evaluation confirming the odontogenic source.
2. Surgical indication established after failure of adequate conservative medical management, defined as persistence of symptoms and endoscopic signs of infection after at least two courses of broad‐spectrum oral antibiotic therapy.
3. Availability of preoperative unenhanced maxillofacial CT imaging.

### 2.2 Exclusion Criteria

Prior or concurrent diagnosis of chronic rhinosinusitis with or without nasal polyps (CRSwNP/CRSsNP).

All patients underwent nasal endoscopy performed by an otolaryngologist, identifying unilateral purulence, edema, or inflammatory changes in the middle meatus or maxillary sinus, in accordance with the diagnostic criteria proposed by Craig et al. Dental evaluation confirmed the odontogenic source in all cases.

All patients presented with sinonasal symptoms—including purulent rhinorrhea, nasal obstruction (unilateral or bilateral), maxillary facial pressure, and postnasal drip—temporally related to prior dental disease or treatment. None of the patients responded to conservative medical management, including topical and systemic corticosteroids, antibiotic therapy resistance, defined as the persistence of symptoms and endoscopic signs of infection after at least two 10 to 14 day cycles of broad‐spectrum oral antibiotics (e.g., amoxicillin/clavulanic acid or fluoroquinolones) [2] and nasal washes. All patients had previously undergone or been offered conservative medical treatment prior to surgical referral. Surgical indication was established only following documented failure of adequate medical management. Then, in patients with reversible odontogenic causes, dental treatment was performed either as the primary intervention or in combination with ESS, according to the multidisciplinary team's assessment.

Three mutually exclusive surgical approaches were adopted: FESS alone, oral surgery alone, and combined surgery, defined as the simultaneous performance of FESS and oral surgery under a single general anesthetic session. No staged procedures, in which ENT and dental interventions were performed in separate operative sessions, were included in this series.

ENT examination is performed with flexible endoscope to examine the sinuses and discover areas of inflammation, displaced dental implants, or anatomical anomalies (nasal turbinate anomalies, nasal polyps, septal deviation). Dentist examination evaluates presence of OAF or dental lesions. A CT scan of the paranasal sinuses without contrast medium was obtained, revealing the location, extent, and severity of the disease, presence of foreign body in maxillary sinus, obstruction of ostiomeatal complex, oro‐antral fistula (OAF). Surgical success was defined as the complete resolution of symptoms and the absence of endoscopic signs of sinusitis at post‐operative controls performed at 10, 30, and 90 days after the intervention [2]. Surgical success was defined as complete or near‐complete resolution of sinonasal symptoms together with the absence of endoscopic signs of active infection or inflammation. Postoperative follow‐up was based on clinical examination and nasal endoscopy. Routine radiological reassessment was not systematically performed, as endoscopic evaluation was considered sufficient in the presence of a surgically created maxillary antrostomy. Dental outcome was considered satisfactory when the odontogenic source had been appropriately addressed, including closure of an oroantral fistula, removal of a foreign body, or control of the odontogenic focus according to the planned multidisciplinary treatment.

### 2.3 Domains Included in the Classification

#### 2.3.1 Timing

The timing component (T) describes the duration of sinonasal symptoms at the time of first ENT evaluation. In accordance with the European Position Paper on Rhinosinusitis (EPOS 2020), we chose as cut‐off for timing 12 weeks. We introduced a third subclass for patients in whom a maxillary sinus foreign body was identified incidentally.

- subclass A: when the symptoms have been present for less than 12 weeks (**a = acute**)
- subclass C: when the symptoms last more than 12 weeks (**c = chronic**)
- subclass 0: when a foreign body at the level of the maxillary sinus can be seen even when the patient does not have any sinusitis symptoms (**0 = without sinusitis**)

The T component of the TEBOF classification reflects the duration of sinonasal symptoms at the time of first specialist evaluation and is intended as a descriptive variable characterizing the patient's clinical presentation, rather than as a parameter directly guiding the timing or type of surgical intervention.

The T_0_ subclass does not represent a diagnosis of odontogenic sinusitis; rather, it identifies a clinically relevant condition in which an asymptomatic maxillary sinus foreign body requires monitoring and, in most cases, removal, consistent with the observations of Gonzales‐Garcia et al. [11].

### 2.4 Etiology

The etiological component of the TEBOF classification (E) was developed building upon the framework originally proposed by Felisati et al. [12] which categorizes ODS according to its relationship with dental disease or implantological treatment. This subdivision was retained and adapted within our system to ensure continuity with established literature while integrating etiology into a broader multidimensional classification.

- “Classical” dental treatment complications—named with capital letter “E”—Subclass E_c_ (“classical”) encompasses sinusitis arising as a direct complication of primary dental pathology including: periapical disease, periodontitis, or post‐extractive complications. This scenario occurs in the absence of any implantological procedure. This subclass represents the most prevalent etiological form in our cohort.
- Implantological treatment complications—Subclass E_a_ (“after implant”) refers to sinusitis developing as a consequence of dental implant placement, whether attributable to implant displacement into the sinus cavity, peri‐implant infection, or intraoperative perforation of the Schneiderian membrane. *In cases where sinus floor elevation and implant placement were performed simultaneously, patients were classified as E*_*a*_.
- Preimplantological treatment complications—Subclass E_p_ (‘before implant’) includes sinusitis arising as a complication of preparatory implantological procedures, most notably sinus floor elevation, performed preparing for implant placement but prior to its execution.

The distinction between post‐implantological (E_a_) and pre‐implantological (E_p_) complications was deliberately maintained in the TEBOF classification, in accordance with the etiological framework proposed by Felisati et al. [12]. These two subclasses differ in their surgical context and in the presence of an indwelling implant as a potential ongoing infectious source, with direct implications for treatment planning. The limited number of Ep patients in the present cohort reflects the epidemiological distribution of this specific etiology in our referral population and does not diminish the clinical relevance of this distinction.

### 2.5 Foreign Body (B = Body)

Records the presence or absence of a foreign body within the maxillary sinus, as detected on CT imaging. Foreign bodies—most commonly displaced dental implants, filling materials, or bone substitute fragments—represent a well‐recognized trigger of mucosal inflammatory response and are classified as B_1_ when present and B_0_ when absent.

### 2.6 Obstruction of Ostiomeatal Complex (O = Obstruction)

The obstruction component (O) reflects the patency status of the ostiomeatal complex (OMC), as assessed on CT imaging and ENT endoscopic evaluation. OMC obstruction impairs physiological sinus drainage and mucociliary clearance, contributing to the persistence and progression of sinusitis. Patients with OMC obstruction are classified as O_1_; those with a patent OMC as O_0_.

### 2.7 Oroantral Fistula (F = Fistula)

The fistula component (F) captures the presence or absence of an oroantral fistula (OAF), defined as a pathological communication between the oral cavity and the maxillary sinus. OAF is identified through clinical examination by the dental provider and represents both a potential source of ongoing sinus contamination and an independent surgical target. Patients with OAF are classified as F_1_; those without as F_0_.

Each factor is identified with a letter, thus obtaining an acronym: TEBOF classification (Table 1).

**TABLE 1:** TEBOF classification system for odontogenic sinusitis. The classification comprises five clinically relevant domains: Timing (T), Etiology (E), presence of a foreign Body (B), Obstruction of the ostiomeatal complex (O), and presence of an oroantral Fistula (F), each further divided into the corresponding subclasses.

Class	Subclass
Timing	acute (less than 12 weeks)
	chronic (more than 12 weeks)
	0: foreign body without sinusitis
Etiology	after implantological treatment complications
	before implantological treatment
	classic complications
Foreign body	0: absence
	1: presence
Obstruction of ostomeatal complex	0: absence
	1: presence
Oroantral fistula	0: absence
	1: presence

Additionally, each letter has a neighboring letter or number that designates a subclass for each factor.

Examples:

- A patient suffering from sinusitis due to a complication of implantological treatment for at least 12 weeks, with OAF, without obstruction of ostiomeatal complex and without foreign body can be identified as: T_c_E_a_B_0_O_0_F_1_ (Figures 1 and 2).
- A patient presenting sinusitis symptoms for less than 12 weeks, after an implantological treatment, without a foreign body in the maxillary sinus, with obstruction of the OMC and with OAF, can be identified as: T_a_E_a_B_0_O_1_F_1_ (Figure 3).
- A patient with chronic sinusitis (more than 12 weeks) as complication of dental pathology, with foreign body in the maxillary sinuses, without obstruction of the OMC and with OAF, can be identified as: T_c_E_c_B_1_O_0_F_1_ (Figure 4).

**FIGURE 1:**
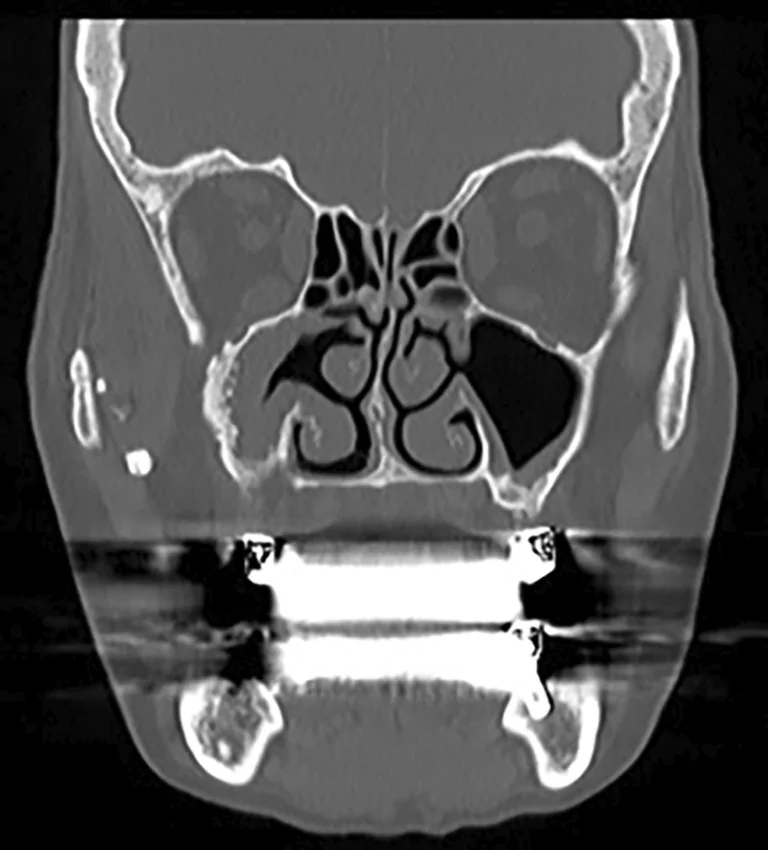
Coronal CT scan of a representative patient classified as T_c_E_a_B_0_O_0_F_1_, showing unilateral left maxillary sinus opacification as a complication of dental implant placement. No foreign body is detected within the sinus cavity, and the ostiomeatal complex is patent. An oroantral fistula was confirmed on clinical examination.

**FIGURE 2:**
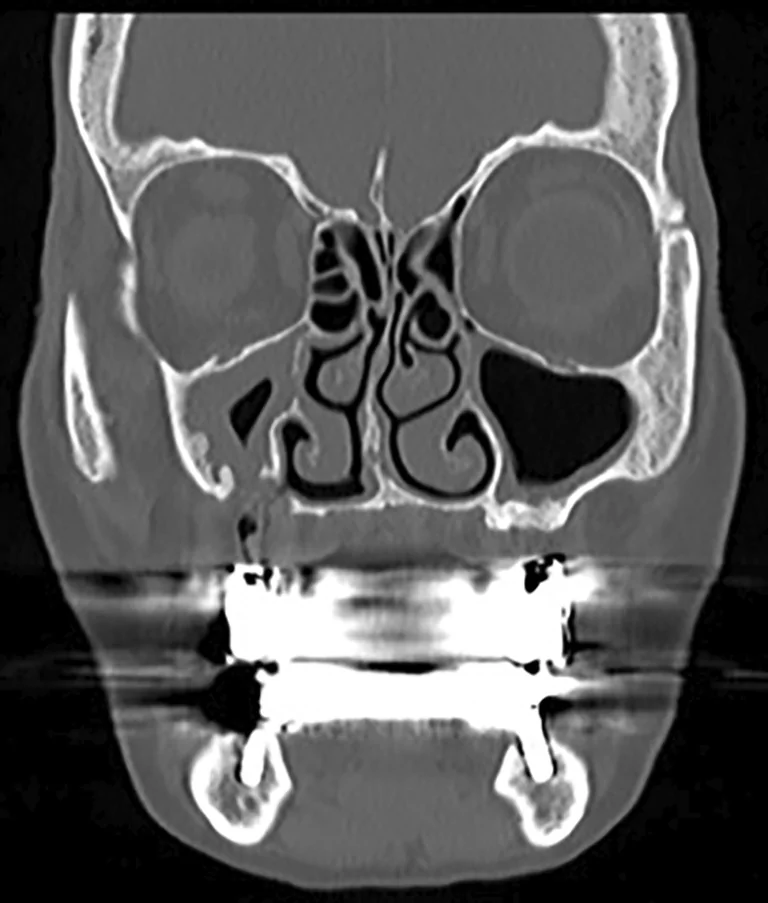
Axial CT scan of the same patient classified as T_c_E_a_B_0_O_0_F_1_, confirming unilateral maxillary sinus mucosal thickening consistent with chronic odontogenic sinusitis following implantological treatment.

**FIGURE 3:**
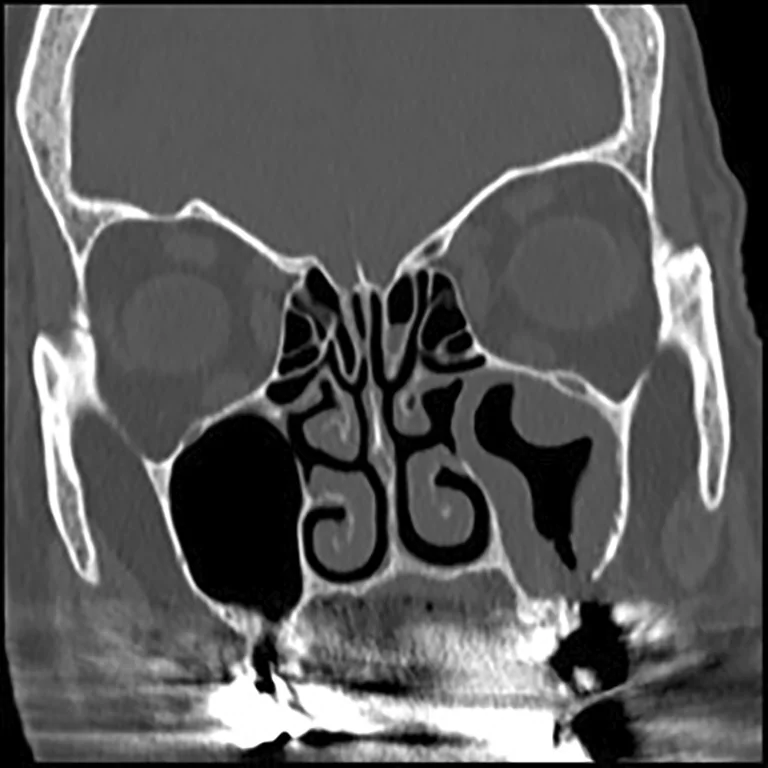
Coronal CT scan of a patient classified as T_a_E_a_B_0_O_1_F_1_, presenting with acute odontogenic sinusitis (symptom duration < 12 weeks) following dental implant placement. The ostiomeatal complex is obstructed and an oroantral fistula is present. No foreign body is identified.

**FIGURE 4:**
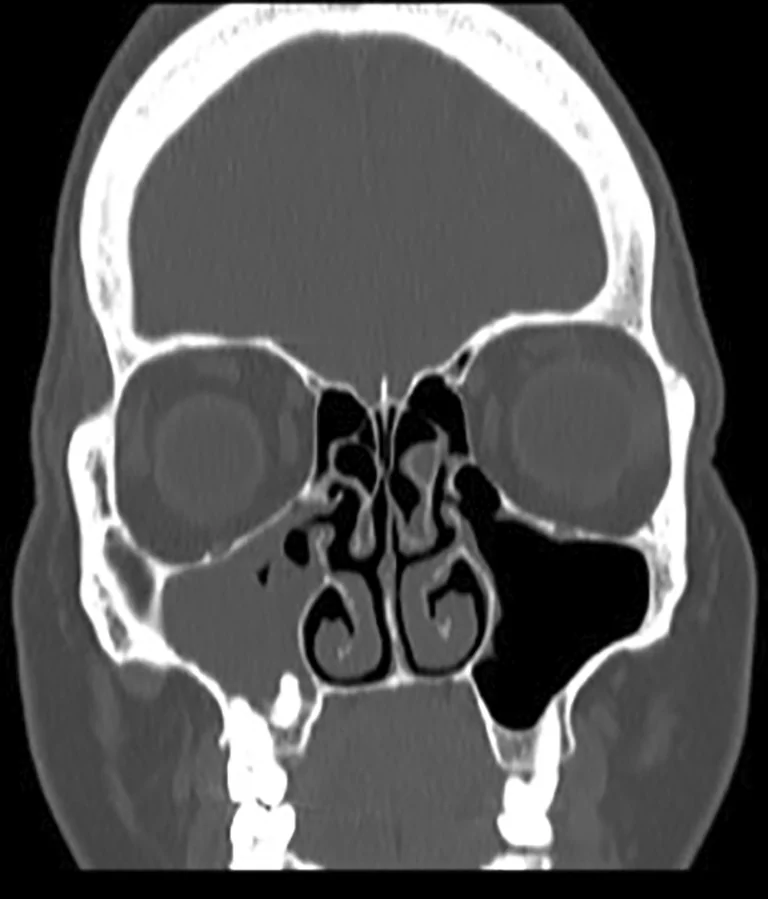
Coronal CT scan of a patient classified as T_c_E_c_B_1_O_0_F_1_, showing chronic odontogenic sinusitis arising from classical dental pathology. A foreign body (displaced dental material) is visible within the maxillary sinus. The ostiomeatal complex is patent and an oroantral fistula is present.

## 3 Ethical Approval

This retrospective observational study was conducted in accordance with the Declaration of Helsinki. As it involved only the review of anonymized clinical records routinely collected during standard care, formal ethics committee approval was not required under applicable institutional and national regulations. Informed consent: All patients provided written informed consent for the use of their anonymized clinical data for research purposes.

## 4 Results

This retrospective study included 124 consecutive patients presenting with clinical and/or radiological signs of maxillary sinusitis, treated at the Department of Otolaryngology, Fondazione Policlinico Universitario Campus Bio‐Medico (Rome, Italy) between December 2020 and December 2023. Patients in whom a maxillary sinus foreign body was identified on CT imaging in the absence of overt sinonasal symptoms were also included, given the well‐established role of foreign bodies as triggers of mucosal inflammatory response and potential precursors of clinically overt sinusitis.

Of the 124 patients, 64 were female (51.6%) and 60 were male (48.4%), yielding a male‐to‐female ratio of 0.94:1. The mean age was 57.2 ± 13 years (range: 26–87 years). All patients reported a history of dental disease or prior oral surgical intervention.

Symptoms' duration was assessed through patient history obtained during the initial ENT or dental evaluation. The majority of patients (96/124, 77.4%) reported symptoms persisting for 12 weeks or longer and were accordingly classified as chronic sinusitis (T_c_ group). Twenty‐three patients (18.5%) presented with a symptom duration of less than 12 weeks and were classified as acute sinusitis (T_a_ group) (Figure 5).

**FIGURE 5:**
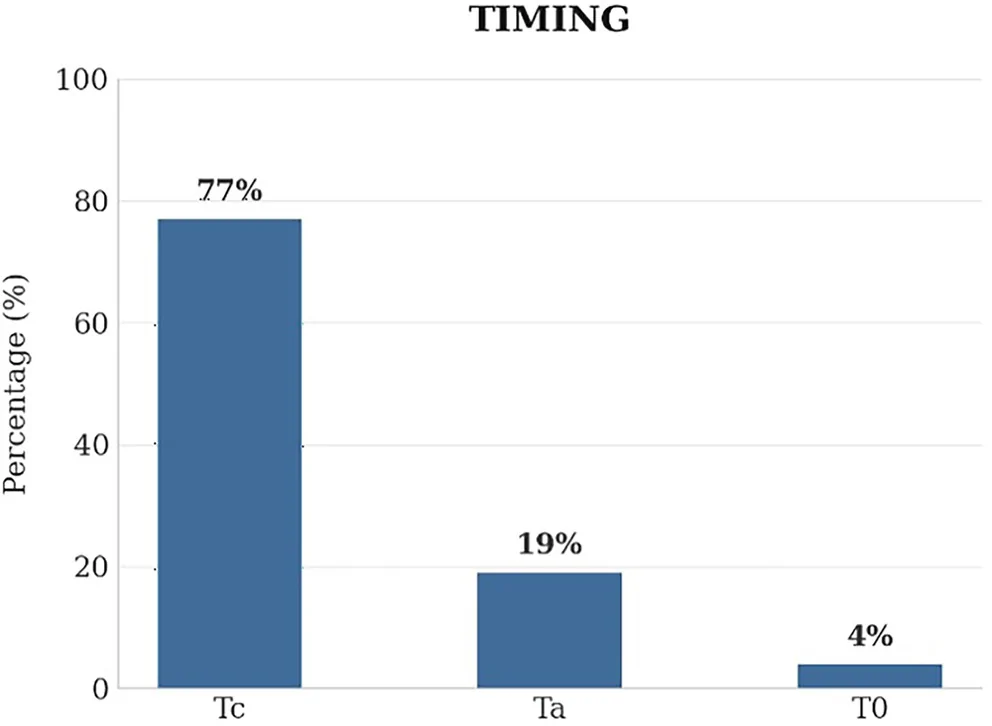
Distribution of the 124 study patients according to the timing (T) component of the TEBOF classification: chronic sinusitis (T_c_, *n* = 96, 77.4%), acute sinusitis (T_a_, *n* = 23, 18.5%), and asymptomatic maxillary sinus foreign body without sinusitis (T_0_, *n* = 5, 4.0%).

A minority of patients (5/124, 4.0%) had no overt sinonasal symptoms at presentation but were found to exhibit a maxillary sinus foreign body on CT imaging; these patients were assigned to the T_0_ subclass.

All patients presented with an identifiable odontogenic etiology. The majority (88, 70.9%) were classified in the E_c_ subgroup, encompassing sinusitis arising as a complication of primary dental pathology. Patients in this subgroup frequently presented to the ENT specialist with sinonasal complaints, often unaware of an underlying dental source of infection. Conversely, 30 patients (24.2%) were assigned to the E_a_ subgroup, having developed sinonasal symptoms following dental implant placement. Six patients (4.8%) were classified in the E_p_ subgroup, in whom sinusitis arose after preparatory implantological procedures, specifically sinus floor elevation, performed prior to implant placement (Figure 6).

**FIGURE 6:**
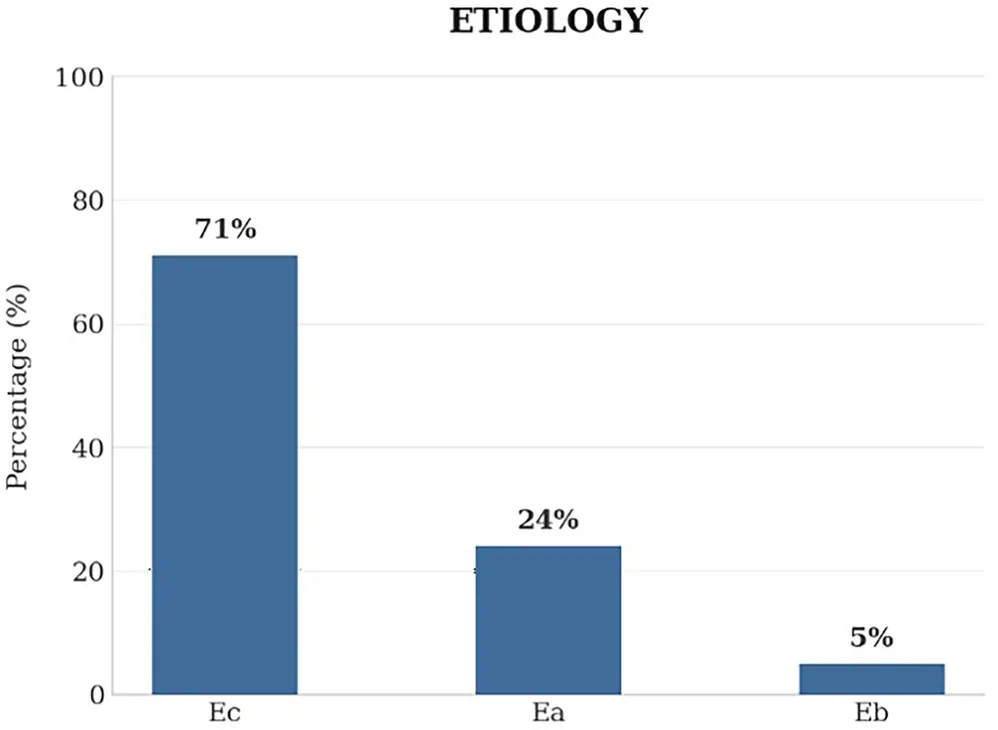
Distribution of the 124 study patients according to the etiology (E) component of the TEBOF classification: classical dental pathology complications (E_c_, *n* = 88, 70.9%), sinusitis after dental implant placement (E_a_, *n* = 30, 24.2%), and sinusitis after pre‐implantological procedures (E_p_, *n* = 6, 4.8%).

Patients belonging to the E_a_ and E_p_ subgroups typically presented to the ENT specialist following an initial dental evaluation, reflecting the implantological context of their condition.

Through clinical examination and imaging, 80 (64.5%) patients with obstruction of the ostiomeatal complex (O_1_ group) were observed (Figure 7).

**FIGURE 7:**
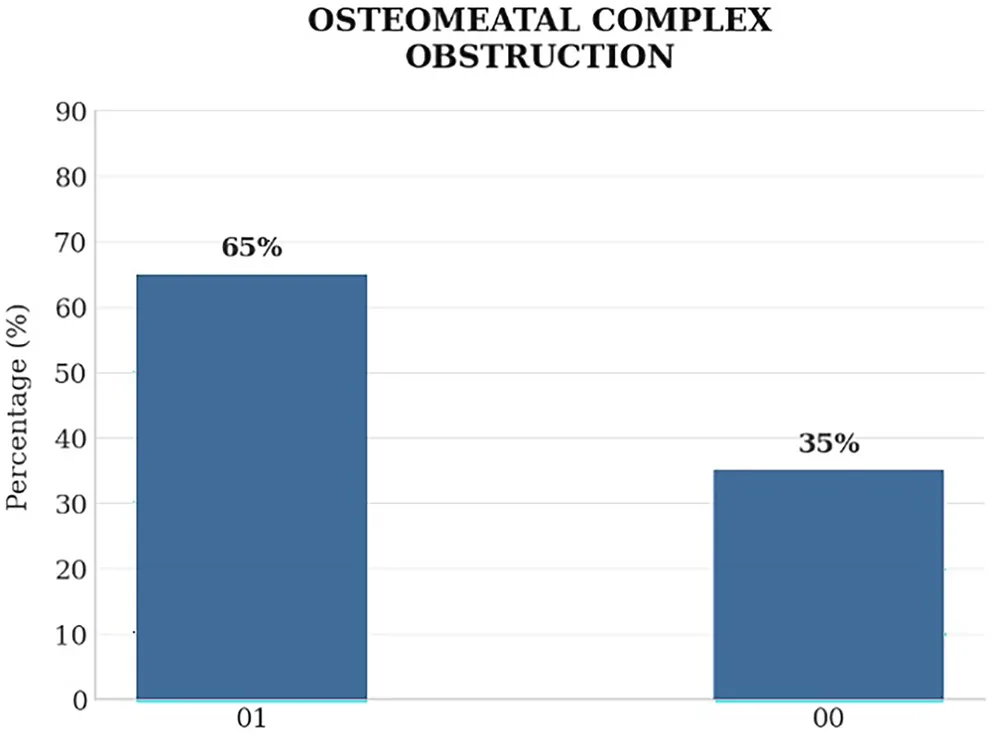
Distribution of the 124 study patients according to the ostiomeatal complex obstruction (O) component of the TEBOF classification: obstruction present (O_1_, *n* = 80, 64.5%) and obstruction absent (O_0_, *n* = 44, 35.5%).

A foreign body was identified in 39 patients (31.5%, B_1_ group), while the remaining 85 patients (68.5%) showed no foreign body (B_0_ group) (Figure 8).

**FIGURE 8:**
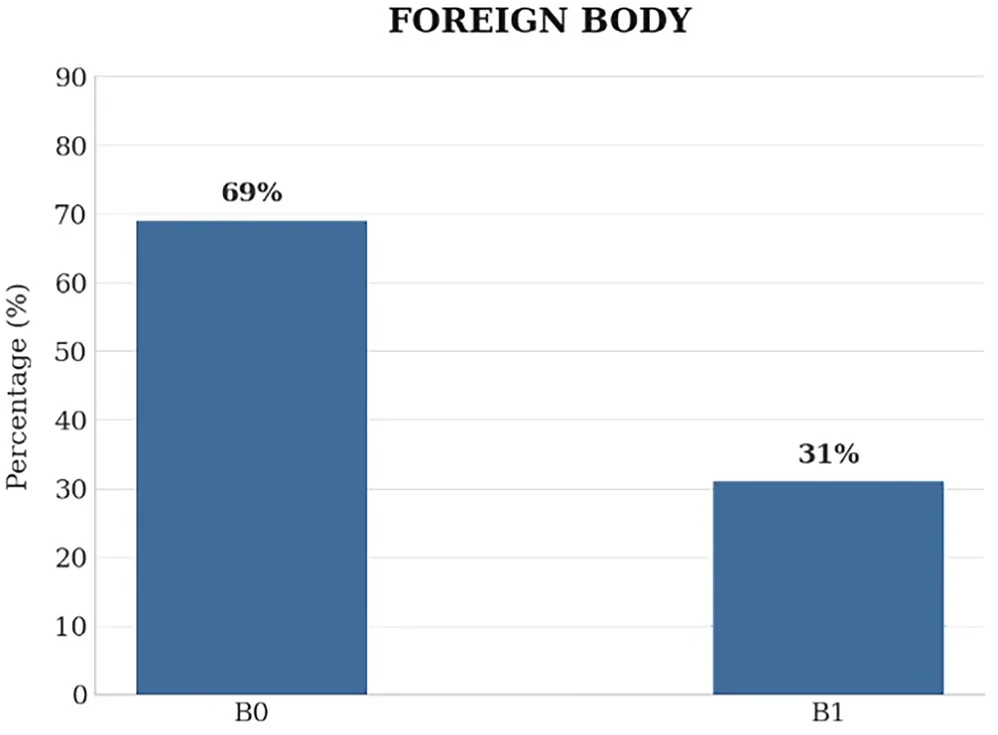
Distribution of the 124 study patients according to the foreign body (B) component of the TEBOF classification: foreign body present (B_1_, *n* = 39, 31.5%) and foreign body absent (B_0_, *n* = 85, 68.5%).

Oroantral fistula was observed in almost half of patients (52, 41.9%) (Figure 9).

**FIGURE 9:**
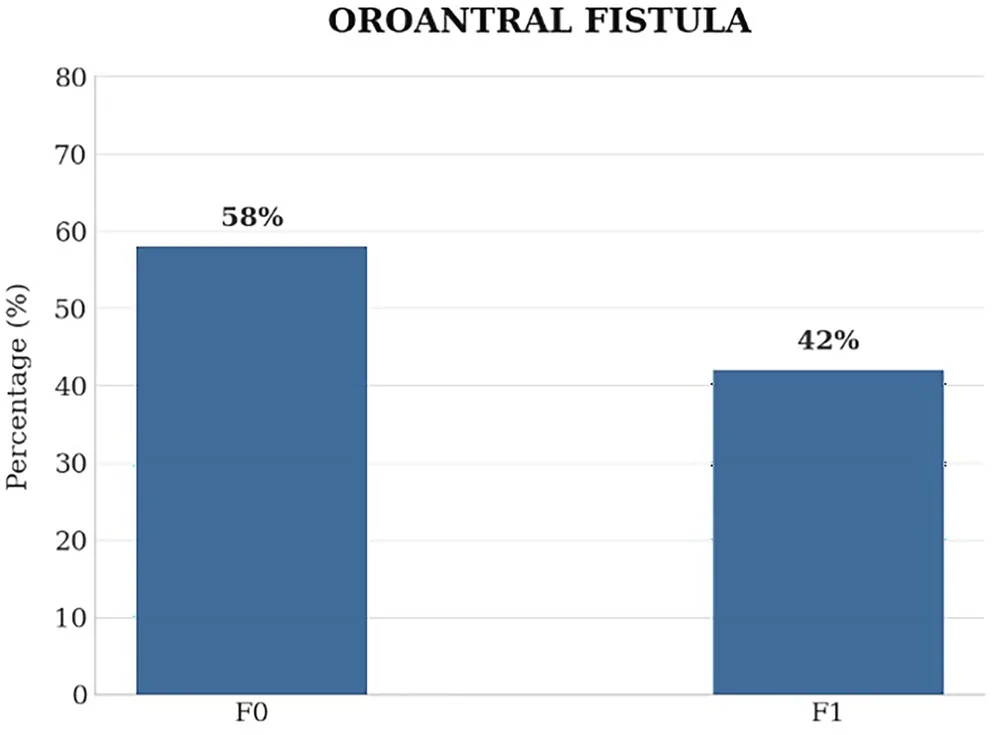
Distribution of the 124 study patients according to the oroantral fistula (F) component of the TEBOF classification: fistula present (F_1_, *n* = 52, 41.9%) and fistula absent (F_0_, *n* = 72, 58.1%).

A patient may have more than one of these conditions at the same time.

The complete TEBOF alphanumeric code was assigned to each of the 124 patients. A total of 31 distinct TEBOF combinations were identified across the cohort, reflecting the marked clinical heterogeneity of odontogenic sinusitis. The three most frequent combinations—**T**_**c**_ **E**_**c**_ **B**_**0**_ **O**_**1**_ **F**_0_ (25 patients, 20.2%), **T**_**c**_ **E**_**c**_ **B**_**0**_ **O**_**1**_ **F**_**1**_ (14 patients, 11.3%), and **T**_**c**_ **E**_**c**_ **B**_**0**_ **O**_**0**_ **F**_**0**_ (13 patients, 10.5%)—together accounted for 52 patients (41.9% of the cohort). The 10 most prevalent TEBOF profiles collectively represented 89 patients (71.8%) of the study population (Table 2).

**TABLE 2:** TEBOF profiles and rate of intervention by type.

TEBOF code	*N*	*N* (%)	Combined (%)	ESS only (%)	Oral only (%)	Predominant approach
T_c_E_c_B_0_O_1_F_0_	25	20.2	64.0%	32.0%	4.0%	Combined
T_c_E_c_B_0_O_1_F_1_	14	11.3	92.9%	0.0%	7.1%	Combined
T_c_E_c_B_0_O_0_F_0_	13	10.5	30.8%	15.4%	53.8%	Oral only
T_c_E_c_B_0_O_0_F_1_	9	7.3	33.3%	0.0%	66.7%	Oral only
T_c_E_a_B_1_O_1_F_1_	7	5.6	100.0%	0.0%	0.0%	Combined
T_c_E_a_B_1_O_1_F_0_	6	4.8	83.3%	16.7%	0.0%	Combined
T_c_E_c_B_1_O_1_F_0_	4	3.2	75.0%	25.0%	0.0%	Combined
T_c_E_c_B_1_O_0_F_0_	4	3.2	25.0%	25.0%	50.0%	Oral only
T_a_E_c_B_0_O_1_F_0_	4	3.2	25.0%	50.0%	25.0%	ESS only
T_a_E_c_B_0_O_1_F_1_	3	2.4	100.0%	0.0%	0.0%	Combined

*Note:* Percentages for intervention type are calculated within each TEBOF profile. Overall surgical success in the cohort was 97% and is reported separately in the results section.

Notably, patients classified as T_c_E_a_B_1_O_1_F_1_ and T_c_E_a_B_1_O_1_F_0_ were exclusively managed with a combined surgical approach, whereas patients with profiles characterized by absent obstruction and absent foreign body and presence of oroantral fistula (T_c_E_c_B_0_O_0_F_1_) were entirely treated with oral intervention alone, suggesting a potential role of the TEBOF code in guiding the selection of a tailored surgical strategy according to the specific alphanumeric profile assigned to each patient.

All patients included in this study had already been treated with medical therapy alone, with persistence of odontogenic sinusitis. Every patient received surgical care. Anatomical features such as the presence of a foreign body, an OAF, and obstruction of the ostiomeatal complex are the main factors used to determine surgical treatment. In the present cohort, odontogenic sinusitis was surgically addressed through three distinct approaches:

- FESS (20, 16%), by ENT specialist alone.
- oral surgery (34, 27%), by the dentist alone.
- combined surgical approach (70, 57%).

Among the 34 patients treated with oral surgery alone, the predominant TEBOF profiles were T_c_E_c_B_0_O_0_F_0_ (*n* = 7) and T_c_E_c_B_0_O_0_F_1_ (*n* = 9). Oral procedures included dental extraction (*n* = 10), endodontic treatment or apicectomy (*n* = 15), and oroantral fistula repair without ESS (*n* = 9). These patients shared the absence of OMC obstruction and foreign body, reflecting the absence of sinonasal surgical targets (Figure 10).

**FIGURE 10:**
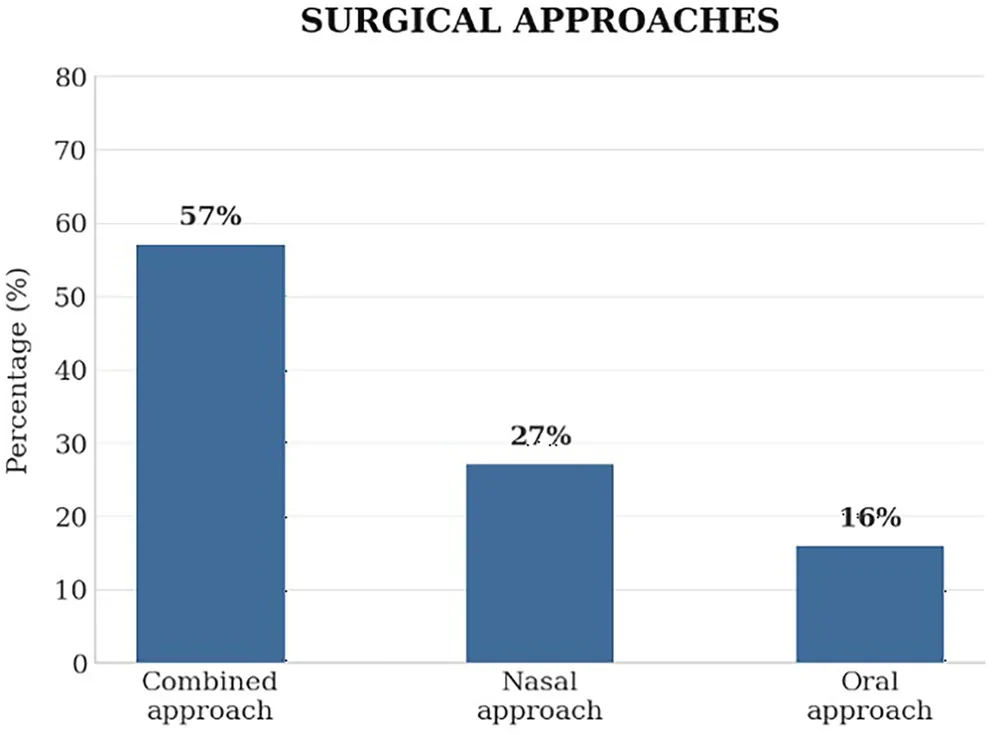
Distribution of the 124 study patients according to the surgical approach adopted: Functional endoscopic sinus surgery alone (ESS, *n* = 20, 16%), oral surgery alone (*n* = 34, 27%), and combined ESS plus oral surgery (*n* = 70, 57%).

This flexibility of methods allows to treat this condition in a tailored fashion.

The overall surgical success rate was 97%, confirming the effectiveness of the multidisciplinary approach adopted for the management of odontogenic sinusitis. Only four patients required a second surgical procedure.

## 5 Discussion

Odontogenic sinusitis represents a clinical challenge owing to its marked heterogeneity. Numerous classification systems have been proposed in the literature, introduced by both otolaryngologists and dental providers, each addressing different aspects of the condition. This study introduces the TEBOF classification system, a multidimensional alphanumeric code designed to characterize odontogenic sinusitis across five clinically relevant domains: timing, etiology, foreign body, ostiomeatal complex obstruction, and oroantral fistula. Applied to a cohort of 124 consecutive patients, the system generated 31 distinct clinical profiles, reflecting the well‐known heterogeneity of ODS and confirming that a single‐variable classification is inherently insufficient to capture the full clinical complexity of this condition. Previous attempts to classify ODS have addressed specific dimensions of the disease in isolation. The system proposed by Felisati et al. [12] organizes patients according to the etiology—preimplantological treatment complications, Implantological treatment complications, “Classic” dental treatment complications—and the relationship with dental implantology, providing a useful framework for treatment planning in implantological contexts but not addressing timing or anatomical complicating factors [12]. The radiological classification by Di Girolamo et al. focuses on the degree and distribution of mucosal thickening on CT imaging, offering guidance on sinus disease burden but not integrating etiological or anatomical features relevant to surgical decision‐making [13].

To our knowledge, TEBOF is the first classification to simultaneously encode timing, etiology, foreign body status, ostiomeatal complex patency, and oroantral fistula into a single compact code, enabling a comprehensive characterization suitable for multidisciplinary communication. The analysis of TEBOF profiles across surgical subgroups revealed clinically coherent patterns.

TEBOF system is firstly conceived as a communication tool. The observed variability in surgical approaches within some TEBOF profiles likely reflects additional patient‐specific factors, including foreign body characteristics, fistula dimensions, and odontogenic source treatability, which were assessed preoperatively but are not encoded in the current version of the classification.

The favorable surgical outcomes observed in this cohort are consistent with success rates reported in the current literature for multidisciplinary ODS management and reflect the effectiveness of the treatment strategies adopted rather than the TEBOF classification per se. The preliminary observation that specific TEBOF profiles cluster within defined surgical pathways provides hypothesis‐generating evidence supporting the system's potential role as a decision‐support tool. Formal validation of TEBOF's ability to stratify surgical risk and guide treatment selection will require prospective multicenter studies with standardized outcome measures.

The preliminary observation that specific TEBOF codes cluster embedded within defined surgical pathways supports the hypothesis that the classification may serve not only as a descriptive tool but also as a decision‐support framework.

The inclusion of the T_0_ subclass specifically accounts for the clinically relevant scenario of asymptomatic maxillary sinus foreign body; in agreement with current literature, the present classification supports the recommendation of removing the foreign body in most cases, even without overt sinonasal symptoms [14].

Although the prognostic significance of the acute/chronic distinction in ODS has not been definitively established, in our experience these two conditions frequently differ in their clinical presentation, disease burden, and therapeutic approach. Acute ODS may require urgent surgical intervention prioritizing symptom relief, whereas chronic ODS more commonly demands a planned, multidisciplinary surgical strategy addressing both sinonasal and dental targets, as suggested by Felisati et al. [12].

Patients with OMC obstruction (O_1_) were predominantly managed with ESS, either alone or in combination with oral surgery. In our cohort, OMC obstruction represented one of the factors contributing to the sinonasal surgical indication, rather than an unconditional indication by itself, in line with the broader multidisciplinary assessment framework proposed in the ODS literature. Conversely, patients with a B_0_O_0_ profile were exclusively treated with oral intervention alone, reflecting the absence of sinonasal anatomical targets requiring surgical rhinological management. The subgroup undergoing combined simultaneous surgery showed a high prevalence of O_1_, OAF, and foreign body co‐occurrence, in agreement with current multidisciplinary consensus recommending combined approaches when both sinonasal and dental surgical targets are present.

Some TEBOF profiles, including the most frequent one, do not correspond to a single uniform treatment approach, reflecting the inherent heterogeneity of the contributing factors—most notably the size and position of the foreign body and the extent of maxillary sinus inflammation.

The favorable surgical outcomes observed in this cohort, consistent with the success rates reported in the current literature, lend further support to the effectiveness of the TEBOF classification in orienting the selection of the most appropriate treatment strategy for each patient.

ODS management inherently requires collaboration between otolaryngologists and dental providers who may adopt different terminologies and prioritize different clinical variables. A standardized alphanumeric code that encodes the most relevant clinical features in a compact, immediately interpretable format can reduce communication errors, facilitate referral, and support consistent documentation in multidisciplinary settings. Some limitations of the present study deserve consideration. The retrospective design and the single‐center setting represent inherent constraints, and the cohort—composed exclusively of patients refractory to medical management—likely reflects a more severe disease spectrum than would be encountered in the general ODS population. Further, it does not provide specific surgical procedural details, as these are necessarily dictated by the individual clinical conditions of each patient—including anatomical variants, size and position of the foreign body, and dimensions of the oroantral fistula—as well as by the surgical expertise of the operating team and the availability of local resources and devices [6]. Nonetheless, this selection mirrors the clinical reality of tertiary ENT referral centers, where the TEBOF system is most likely to find application. These findings suggest that the systematic characterization of ODS through the TEBOF classification closely reflects the selection of the most appropriate therapeutic strategy for each patient. Future prospective studies in multicenter settings will be instrumental in evaluating the inter‐rater reliability of TEBOF assignment and its correlation with surgical outcomes, potentially extending its role from a descriptive to a predictive tool.

## 6 Conclusions

The TEBOF classification system provides a concise, reproducible, and clinically intuitive framework for the characterization of odontogenic sinusitis. Its five components are assessable through standard clinical workup: anamnesis defines timing and etiology [15]; ENT and dental examination identifies sinusitis signs and oroantral fistula, and CT imaging reveals foreign body presence and ostiomeatal complex status [16].

This compact alphanumeric code allows different specialists to rapidly exchange the pivotal clinical information required for the appropriate multidisciplinary management of ODS [17].

The results of the present study provide encouraging preliminary support for the clinical utility of the TEBOF classification, suggesting that a structured phenotyping of odontogenic sinusitis may meaningfully contribute to the selection of the most appropriate therapeutic strategy. Future prospective multicenter studies will be essential to validate these findings and to consolidate the role of the TEBOF system as a treatment‐guiding tool in the multidisciplinary management of odontogenic sinusitis.

## Funding

The authors have nothing to report.

## Disclosure

During the preparation of this manuscript, the authors used Gemini (Large Language Model, Google, Mountain View, CA, USA) for the purposes of English language editing and proofreading. The authors have reviewed and edited the output and take full responsibility for the factual accuracy and original content of this publication.

## Consent

Routine general written informed consent for clinical and surgical procedures, including the potential use of anonymized medical records for research purposes, was obtained from all patients at the time of treatment.

## Conflicts of Interest

The authors declare no conflicts of interest.

## Data Availability

The data that support the findings of this study are available from the corresponding author upon reasonable request.
